# Editorial: Advances in mast cell physiology and mast cell-driven diseases

**DOI:** 10.3389/fimmu.2023.1303726

**Published:** 2023-10-19

**Authors:** Ulrich Blank, Carlo Pucillo

**Affiliations:** ^1^ Université Paris Cité, Centre de Recherche sur l’Inflammation, Institut National de la Santé et de la Recherche Médicale (INSERM) Unité Mixte de Recherche (UMR)1149, Centre National de la Recherche Scientifique (CNRS) Equipe Mixte de Recherche (EMR)-8252, Faculté de Médecine site Bichat, Paris, France; ^2^ Université Paris Cité, Laboratoire d’Excellence INFLAMEX, Paris, France; ^3^ Department of Medicine, University of Udine, Udine, Italy

**Keywords:** mast cell, inflammation, allergies, pseudo-allergies, inflammatory mediators, mastocytosis

Mast cells (MCs) are long-lived tissue-resident immune cells occupying their final residence in several locations, including the fetal liver during fetal development. In adults, they arise from bone marrow–derived progenitors, which, after a short passage in the blood, migrate to their final destination and differentiate into mature MCs (1). They are predominant in tissues that are in close contact with the external environment, such as the skin or at mucosal surfaces, but can be found in virtually every organ, often increasing during an inflammatory process (2).

They are well-known effectors of IgE-mediated immune responses protecting against parasitic infections and toxins such as snake venoms but are also key effectors of allergies. When activated through IgE receptors (FcεRI), they release a whole set of inflammatory mediators stored in cytoplasmic granules, including histamine and various MC-specific proteases (3). This is followed by a wave of newly synthesized lipid mediators, such as prostaglandins and leukotrienes, and a large variety of chemokines and cytokines, thereby perpetuating and regulating the initiated inflammatory response. Besides FcεRI, MCs express a large set of other receptors (4) empowering them to respond to IgE-independent stimuli in various physiological settings, implicating them as contributors to IgE-independent autoimmune diseases, various chronic inflammatory disorders, and cancers including mastocytosis. A recently described receptor, MRPRX2, has received great attention due to its ability to interact with a large variety of positively charged chemical compound and peptides, being responsible for the development of so-called pseudo-allergic reactions as well as the crosstalk with the peripheral nervous system, releasing neuropeptides (5).

This Research Topic assembles 10 original research articles and one review article with a large majority (five) devoted to the recently discovered MC-specific receptor MRGPRX2. Two articles analyze MCs in the context of transformation and mastocytosis, and the remaining articles seek to further characterize and describe the physiology/pathophysiology of MCs in different contexts. Concerning the study of MRGPRX2, Thapaliya et al. investigate the effect of various inhibitors of G protein-coupled receptor (GPCR) kinase 2 (GRK2) on IgE- and MRGPRX2-initiated MC responses. The authors show that while these inhibitors readily inhibit IgE-induced anaphylactic responses, they have a contrary activating role on human MRGBRX2-induced responses and its murine counterpart MRGPRB2. Conversely, Bawazir et al. show that two inverse MRGPRX2 agonists, while inhibiting the MRGBRX2 agonist responses, did not do so when using the murine equivalent MRGPRP2 or FcεRI-induced responses, setting a framework for selective treatment of IgE-independent MC-mediated drug hypersensitivity and cutaneous disorders. The study by Guo et al. describes a novel signaling pathway initiated by MRGPRX2 and involving nuclear translocation of the moonlighting protein lysyl t-RNA synthetase (LysRS), thereby activating the microphthalmia-associated transcription factor (MITF) involved in various MRGPRX2-initiated MC responses. Raj et al. perform a structural study examining the ability of certain analogs of Substance P to activate MRGPRX2 based on computational studies. The authors confirm the importance of basic residues for full-blown activation, although some responses, such as CCL2 chemokine secretion, can still be detected. Another study by Thapaliya and Ali on the role of GRK2, a Serine/Threonine kinase that promotes desensitization and internalization of GPCRs. Using MCs where this kinase has been specifically knocked out, the authors show that certain but not all FcεRI-initiated responses are inhibited, while, on the contrary, MRGPRB2-initated responses are enhanced, although this was dependent on the nature of the ligand, supporting a role in GPCR receptor desensitization. The authors also reveal a GRK2-independent crosstalk between FcεRI and MRGPRP2. Concerning mastocytosis, Bandara et al. investigate the role of two oncogenic mutations (D816V and V560G) of the KIT receptor by engineering a human MC line, HMC-1.2 cells, with a single D816V-KIT mutation and comparing it to the parent cell line bearing both mutations. The authors reveal several differences and suggest that the cell line with the single D816V-KIT mutation, which is the mutation present in most mastocytosis cases, represents an improved clinical mastocytosis model. Capellmann et al. characterize a new spontaneously developed transformed murine peritoneal MC line with accelerated cell cycle progression, called PMC-306, although the culture was still dependent on the presence of the growth factors IL-3 and SCF. Transformation was accompanied by the loss of the critical cell cycle regulators Cdkn2a and Arf expression that involves the activation of Kit. This new cell line represents a new tool to study MC biology and MC tumorigenic processes. Concerning MC functions, Herrera-Heredia et al. present a study on the physiological role of heparin stored in cytoplasmic granules. Generating a novel experimental model of Heparin deficiency in connective tissue type MCs, the authors present a variety of results highlighting the role of heparin in secretory granule formation and various physiological responses. The role of microgravity, i.e., the complete or near-complete absence of the sensation of weight, on MC functions was studied by Kim et al. using a rotary culture system that simulates microgravity. The authors reveal a variety of negative regulatory functions contributing to the understanding of immune system function/dysfunction in space medicine research. Rönnberg et al. investigate the heterogeneity of sorted human lung MCs implicated in airway inflammatory diseases, such as asthma, using single cell RNA sequencing. The authors reveal a high expression of classical MC markers. Although variable expression of several individual genes could be detected, no specific subpopulation could be detected by unbiased clustering. Finally, Schulman et al. review the role of P2 purinergic cell-surface receptors (P2R) interacting with extracellular adenosine 5′-triphosphate (ATP). ATP is a component of the damage-associated molecular patterns that can potently enhance FcεRI-induced degranulation. Potential therapeutic approaches targeting this interaction are discussed.

Taken as a whole, this Research Topic of original articles gives us new insights into MC functions. A large proportion of the articles concerns the role of MRGPRX2 specifically expressed by MC. They also provide new data on MC biology and models to study MC transformation. We hope that these new insights will contribute to a better understanding of MC physiology/pathophysiology and will, at least partly, also provide new ideas for therapeutic interventions of MC driven diseases.

## Author contributions

UB: Writing – original draft. CP: Writing – original draft.
